# Microbial Diversity of a Brazilian Coastal Region Influenced by an Upwelling System and Anthropogenic Activity

**DOI:** 10.1371/journal.pone.0016553

**Published:** 2011-01-27

**Authors:** Juliano C. Cury, Fabio V. Araujo, Sergio A. Coelho-Souza, Raquel S. Peixoto, Joana A. L. Oliveira, Henrique F. Santos, Alberto M. R. Dávila, Alexandre S. Rosado

**Affiliations:** 1 Laboratory of Molecular Microbial Ecology, UFRJ, Rio de Janeiro, Brazil; 2 Laboratory of Microbiology, DCIEN, FFP/UERJ, Rio de Janeiro, Brazil; 3 INPeTAm/IBCCF, UFRJ, Rio de Janeiro, Brazil; 4 Laboratory of Computational Biology and Systems, IOC, FIOCRUZ, Rio de Janeiro, Brazil; Argonne National Laboratory, United States of America

## Abstract

**Background:**

Upwelling systems are characterised by an intense primary biomass production in the surface (warmest) water after the outcrop of the bottom (coldest) water, which is rich in nutrients. Although it is known that the microbial assemblage plays an important role in the food chain of marine systems and that the upwelling systems that occur in southwest Brazil drive the complex dynamics of the food chain, little is known about the microbial composition present in this region.

**Methodology/Principal Findings:**

We carried out a molecular survey based on SSU rRNA gene from the three domains of the phylogenetic tree of life present in a tropical upwelling region (Arraial do Cabo, Rio de Janeiro, Brazil). The aim was to analyse the horizontal and vertical variations of the microbial composition in two geographically close areas influenced by anthropogenic activity (sewage disposal/port activity) and upwelling phenomena, respectively. A lower estimated diversity of microorganisms of the three domains of the phylogenetic tree of life was found in the water of the area influenced by anthropogenic activity compared to the area influenced by upwelling phenomena. We observed a heterogenic distribution of the relative abundance of taxonomic groups, especially in the Archaea and Eukarya domains. The bacterial community was dominated by Proteobacteria, Cyanobacteria and Bacteroidetes phyla, whereas the microeukaryotic community was dominated by Metazoa, Fungi, Alveolata and Stramenopile. The estimated archaeal diversity was the lowest of the three domains and was dominated by uncharacterised marine Crenarchaeota that were most closely related to Marine Group I.

**Conclusions/Significance:**

The variety of conditions and the presence of different microbial assemblages indicated that the area of Arraial do Cabo can be used as a model for detailed studies that contemplate the correlation between pollution-indicating parameters and the depletion of microbial diversity in areas close to anthropogenic activity; functional roles and geochemical processes; phylogeny of the uncharacterised diversity; and seasonal variations of the microbial assemblages.

## Introduction

The coastal upwelling consists of an upward movement of bottom seawater and depends on the confluence of meteorological factors and continental morphology. Upwelling systems are characterised by an intense primary biomass production in the surface (warmest) water after the outcrop of the bottom (coldest) water, which is rich in nutrients. The Arabian Sea and coasts of Namibia, Chile, Peru and Benguela display areas where upwelling phenomena occur in a more pronounced way [1]–[4]. Some studies have confirmed the existence of a typical upwelling system on the southeast Brazilian coast and that the Arraial do Cabo region is the location where the main event occurs [5]–[7]. In this region, the South Atlantic Central Water (SACW) rises to the surface of the ocean in accordance with the east-northeast winds that occur more frequently between spring and summer and also due to the influence of cyclonic meanders [8]–[11].

Studies have demonstrated the ecological importance of these systems, with nitrogen loss by nitrification [1], [12], [13] and increased biological productivity caused by the outcropping of the deep waters [14]–[17] stimulating fishing activities [18].

Although it is known that the microbial assemblage plays an important role in the food chain of marine systems [19] and that the Brazilian upwelling system drives the complex dynamics of the food chain [20], little is known about the microbial composition present in the central area of such upwelling. Moreover, the microbial composition of a region can vary due factors such as ocean water warming [21], water column depth [22], water mass boundaries [23] and the presence of pollutants [24]. Furthermore, there is little information available concerning the microbial diversity of the Brazilian coast. Culture-independent studies based on SSU rRNA sequences have shown that coastal systems harbour a vast and uncharacterised diversity of microorganisms representing the three domains of the phylogenetic tree of life [25]–[28]. We carried out a molecular survey with the aim of assessing microbial assemblages and determining how their variation in the marine waters of Arraial do Cabo are influenced by upwelling events [29] and anthropogenic activities [30].

## Materials and Methods

### Location, sampling and environmental parameters

**Figure 1:**
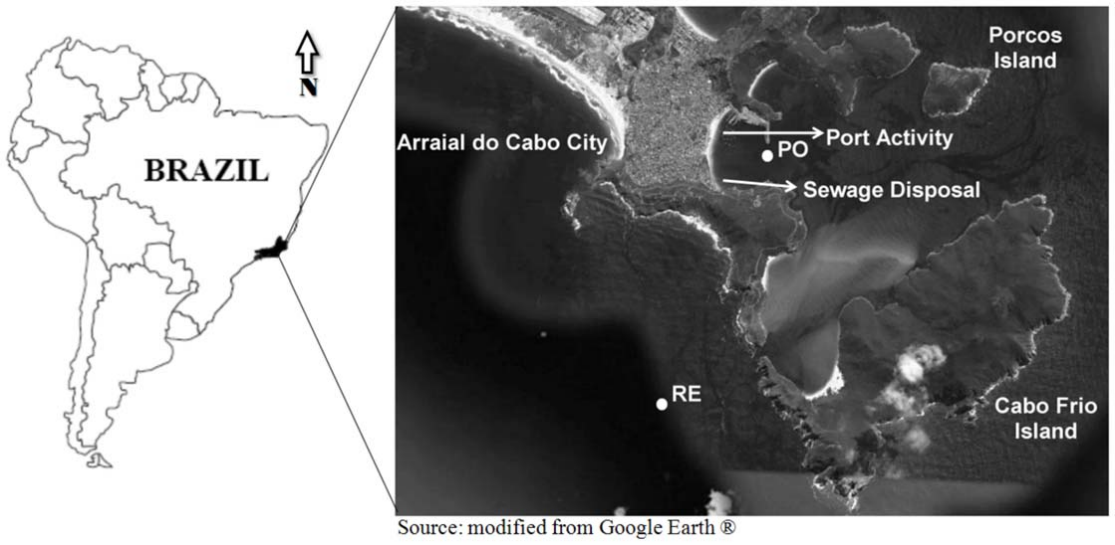
Sampling location. Coastal area of Arraial do Cabo, Cabo Frio region, Rio de Janeiro state, Brazil. White spots indicate the position of sampling sites where water was collected. PO: region influenced by port activity and sewage disposal. RE: open ocean region influenced by upwelling. S: surface. F: bottom (20 m for PO and 50 m for RE).

The studied site is located in Arraial do Cabo, at the Cabo Frio upwelling region, Rio de Janeiro state, Brazil (Fig. 1). Two sites were chosen for the water sampling: one site is located in Baía dos Anjos and is more influenced by anthropogenic activity and less by upwelling (PO); the other site is located in an oceanic area where upwelling (RE) occur in the absence of the influence of anthropogenic activities. Physical-chemical parameters (dissolved oxygen – DO, pH, water temperature, salinity, turbidity and conductivity) were measured *in situ* with a multiparameter probe (YSI, Model). Water samples were collected on 29 February 2008, with a 5-L Van Dorn bottle at the sub-surface (0.5 m) (POS and RES) and at the bottom at depths of 20 m (POF) and 50 m (REF) for PO and RE, respectively. Sub-samples of 100 mL were collected for microbiological counts (coliforms and heterotrophic bacteria), placed in sterilised bags and stored at 4°C on wet ice. For nutrient analysis (ammonia, nitrite, nitrate and phosphate), 1 L of each van Dorn sample was placed in a polypropylene bottle and stored at 4°C on wet ice. For molecular analysis, 1 L of water was collected in triplicate (three van Dorn casts for each sub-surface and bottom depth sampling), placed in autoclaved polypropylene bottles and stored at 4°C on wet ice. All samples stored on wet ice were immediately transported under refrigeration to the laboratory for processing. Twenty microlitres of water from each Van Dorn bottle was separated for bacterial activity and flow cytometry analyses. For bacterial enumeration, three 1.7-mL samples of local water were fixed with 2% paraformaldehyde and frozen. Bacterial concentrations varied around 50% between the samples, but all three samples were pooled before dye input. Volumes of 1.7 mL of sampled water were used for bacterial activity analysis as well. The first step, the addition of ^3^H-leucine, was performed under field conditions in triplicate and using a control. After incubation, the samples were frozen until protein extraction.

### Bacterial counting

Total and thermotolerant coliforms were identified only in sub-surface waters using MPN methods. Serial dilutions of the water samples (10; 1; 0.1; 0.01 and 0.001 mL) were inoculated in lauryl sulphate broth and incubated for 24 h at 35–37°C to determine the total coliform counts. Positive tubes (presence of bubbles and gas production) were reinoculated in tubes containing EC broth and incubated for 48 h at 44.5°C to determine the thermotolerant coliform counts. MPN tables were used to determine the numbers of total and thermotolerant coliforms [31]. The total numbers of heterotrophic bacteria were determined by the spread plate method. Aliquots of 0.1 mL and 10× and 100× dilutions were spread onto plates containing Marine Agar. These plates were incubated at 25±3°C for 48 h, after which the CFUs (colony-forming units) were counted [31].

### Flow cytometry and bacterial activity

Bacterial concentrations were measured with a CytoSense-Cytobuoy flow cytometry equipped with a solid blue laser providing 20 mV at 488 nm, one side scatter (SWS, 446/500 nm) detector and three others to red (chlorophyll-a) fluorescence (FL1- 669/725 nm); orange/yellow (FL-2, 601/651 nm), and green/yellow (FL-3, 515/585 nm) fluorescence, respectively [32]. A yellow-green 0.92 and 10 µm beads (Fluoresbrite Microparticles, Polysciences Inc.-Warrington, PA) were used as an internal standard and to bacterial counts field determination using length sidescatter and average orange fluorescence parameters. Bacterial pooled samples were incubated for 15–30 minutes in the dark with Syber Green I at a final concentration of 0.5×10^−4^ of the commercial stock solution [33]–[35]. Samples were run 4 times for 1 minute at a rate of 2 m.s^−1^ and the discriminator was set to sidescatter and orange fluorescence.

Bacterial activity was assessed according to Smith & Azam [36]. Triplicate samples (1.7 ml) were incubated for 1 h in 2-ml Eppendorf tubes containing L-[4,5-^3^H] leucine (Amersham TRK 510; specific activity: 73 Ci nmol^−1^) at a final concentration of 10 nM. The incubations were performed in situ inside thermal boxes to maintain all samples at the same temperatures. One tube was amended with 90 µl of 100% ice-cold trichloroacetic acid (TCA) as a killed control. After 1 h, the incubation was halted by the addition of TCA, and the tubes were frozen. In the laboratory, samples were processed for ^3^H-protein extraction by centrifugation, and the isotopic activity was determined using a Packard Tricarb 1600 TR liquid scintillation counter with internal calibration and quench correction. Bacterial carbon production was calculated using a protein/carbon conversion factor of 0.86 [37], as used in others studies conducted in this region [20], [38].

### DNA extraction

Each 1-L sample was filtered through a 0.22-µm diameter cellulose filter membrane (Millipore™) and stored at −80°C. Each membrane was macerated with liquid nitrogen and used for DNA extraction. The metagenomic DNA was extracted using the UltraClean™ Soil DNA kit (MOBIO Laboratories – Carlsbad, California) according to the manufacturer's instructions. DNA integrity was verified by electrophoresis in a 1% agarose gel in TAE buffer.

### PCR-DGGE analysis

For PCR-DGGE (denaturing gradient gel electrophoresis) analysis of the bacterial community, the 16S rRNA fragments were amplified by PCR using specific primers (Table 1). The samples were collected in triplicate. DGGE of 40 µl of amplicons of the bacterial 16S rRNA from each sample was carried out using a DCode system (BioRad) at 175 V and 60°C for 16 h in 1X TAE buffer. The 6% (w/v) polyacrylamide gels were made with a denaturing gradient ranging from 30% to 65%. After electrophoresis, the gels were stained with SYBR Green I (Molecular Probes) for 40 min and then scanned using a Storm PhosphorImager (Amersham Biosciences). The dendrograms were constructed after image capture and analysis by Pearson's correlation coefficients (r), and cluster analysis was performed by the unweighted pair group method with average linkages (UPGMA) using BioNumerics software (Applied Maths, Ghent, Belgium). Each band was identified, and its intensity was measured. The band intensity was then expressed as a proportion of the total intensity of all bands comprising a particular community profile.

**Table 1:** Overview of the primers and PCR conditions used to amplify the partial SSU rRNA gene for marine water samples of Arraial do Cabo region.

Primer name	Sequence (5′-3′)*	Used for	PCR Conditions	Ref.
U968F*	AACGCGAAGAACCTTAC	DGGE	3 min initial denaturation at 94°C; 35 cycles of denaturation (1 min at 94°C), annealing (1 min at 55°C), and extention (1 min at 72°C); final extention at 72°C for 10 min	[39]
L1401R	GCGTGTGTACAAGACCC			
BAC27F	AGAGTTTGATCMTGGCTCAG	Library	5 min initial denaturation at 95°C; 30 cycles of denaturation (1 min at 92°C), annealing (1 min at 55°C), and extention (1 min at 72°C); final extention at 72°C for 10 min	[40]
BAC518R	ATTACCGCGGCTGCTGG			
ARCH21F	TTCYGGTTGATCCYGCCIGA	Library	5 min initial denaturation at 95°C; 30 cycles of denaturation (30 s at 95°C), annealing (30 s at 53°C), and extention (1 min at 72°C); final extention at 72°C for 6 min	[41], [42]
ARCH958R	YCCGGCGTTGA(I/C)TCCAATT			
ARCH519R	TTACCGCGGCKGCTG			
EK7F*	ACCTGGTTGATCCTGCCAG	Library/	5 min initial denaturation at 95°C; 30 cycles of denaturation (1 min s at 95°C), annealing (1 min at 55°C), and extention (1 min at 72°C); final extention at 72°C for 10 min	[43], [44]
EK516R	ACCAGACTTGCCCTCC	DGGE		

*For DGGE analysis a GC-clamp (5′-GCCCCCCGCGCCCCGCGCCCGGCCCGCCGCCCCCGCCCC-3′) was added to the 5′ end of the forward primers.

### Construction of SSU rRNA gene clone libraries and sequencing

One sample of each triplicate series of samples (repetition number one) was used for SSU rRNA library construction and sequencing. Table 1 shows the name, sequence, PCR conditions and reference for the primers used. For the 16S rRNA from Archaea, nested PCR was performed using the primer pairs ARCH21F-ARCH958R and ARCH21F-ARCH519R for the first and second amplification, respectively. The amplified fragments were ∼310, ∼530 and ∼450 base pairs in length for the Archaea, Eukarya and Bacteria domains, respectively. Agarose gel electrophoresis of 150 µl of each PCR product was performed prior to purification with the QIAquick Gel Extraction Kit (Qiagen) according to the manufacturer's instructions. Purified amplicons were ligated into the pGEM® T Easy Vector plasmid (Promega). The ligation products were transformed into DH5-α *Escherichia coli* competent cells. Positive clones were grown in LB medium, and the plasmids were isolated using a miniprep alkaline lysis method [45]. Each insert was sequenced using the BigDye terminator system and an ABI-3730 automatic capillary sequencer (Applied Biosystems).

### Sequence analysis

The electropherogram sequencing files were processed using the Phred program [46] for base calling and for trimming of vector and low quality (<20) sequences. The high quality sequences located between the rRNA primers were used for further analysis. The prokaryotic sequences were chimera-checked using the Mallard program [47], and the putative chimeras were excluded from further analysis. Valid sequences were then aligned using ClustalX 1.81 [48]. The PHYLIP format output alignments were used to construct distance matrices within each library using DNADIST provided in the PHYLIP 3.6 package [49], with default parameters and using the Jukes-Cantor model [50] option. The generated matrices were used as input files for DOTUR [51] to calculate the species richness using Chao1 [52] and ACE [53] estimators, the rarefaction curves and the Shannon-Weaver diversity index [54]. The Good's coverage estimator was used to calculate the sample coverage using the formula C = 1-(*n*i/N)×100, where N =  total number of sequences analysed and *n*i  =  number of reads that occurred only once among the total number of reads analysed using DOTUR_0.03_ [55], [56]. The Bacteria and Archaea phyla composition were determined by taxonomic assignment using the RDP Classifier [57] with default parameters through the web service provided by RDP II [58]. For the 18S rRNA sequences, the taxonomic affiliation was determined using the BLAST program [59] provided by NCBI (http://www.ncbi.nlm.nih.gov/).

Fast UniFrac [60] analysis was performed to compare the libraries based on phylogenetic information. The Greengenes core set (May 2009) was used as the source of the reference sequences and the reference tree for the MegaBLAST search and the phylogenetic distribution, respectively. The P Test option was used to test whether each pair of samples was significantly different. The P Test significance (p-values) for each pair of sample comparisons was obtained using 1,000 permutations.

For tree construction, the OTUs (Operational Taxonomic Units) of all sequences were determined together for each domain, as described above for each library. One representative sequence of each OTU was randomly selected for use in the alignments. The nearest-neighbour sequences employed for the construction of the previous trees were obtained using selected representatives of each OTU and the Aligner tool provided by the SILVA database [61]. The generated FASTA file was edited to eliminate redundancy, and the sequences were realigned and manually edited with the ClustalW aligner available in the MEGA 4.0 program [62]. Phylogenetic trees were constructed and edited using the MEGA 4.0 program with the neighbour-joining method, Jukes-Cantor model [50] option and a bootstrap value of 1000.

### Nucleotide sequence accession numbers

The sequences generated were deposited in the GenBank under the accession numbers HM224563 to HM228083.

## Results

### Physical, chemical and biological parameters

Physical-chemical data are presented in Table 2 and demonstrate the spatial distribution of the microorganisms. The samples were collected during a season in which upwelling commonly occurs. As indicated in Table 2, the lowest temperature (below 18°C) and the highest nitrate concentration confirmed the presence of SACW in deep water samples from upwelling area (REF) [6]. The water temperature was 4.5–7°C lower in the REF samples compared to the other sampling places (POS, POF and RES). The pH values were higher in the RES and REF samples compared to the area under more anthropogenic influence (Baía dos Anjos, samples POS and POF). The levels of phosphate and ammonium were lower in REF sample compared to the other three samples, whereas the levels of nitrate and nitrite were higher in REF sample compared to the other three samples.

**Table 2:** Physical, chemical and biological characteristics of the marine water samples of Arraial do Cabo region used in the molecular analysis.

Sample	Dep.(m)	T.(°C)	pH	O_2_(µM)	Sal.(S)	Tot. Coliforms(MPN.100 mL^−1^)	Thermotolerant Coliforms(MPN.100 mL^−1^)	Tot. Heterot. Bacteria(CFU.mL^−1^)	Total Bacteria*(part. mL^−1^)	Bacterial Production(µC L^−1^ h^−1^)	NH4^+^(µM)	Nitrite(µM)	Nitrate(µM)	Phosphate(µM)	N(µM)	P(µM)
POS	0,5	24,5	7.5	281	36,0	2,92	1,41	4,75	1,5×10^5^	0,93±0,15	0.90	0.04	13.00	0.98	15.73	0.98
POF	15	23,0	8,0	263	38,0	N.D.	N.D.	3,08	1,2×10^5^	0,01±0,00	1.03	0.06	16.51	0.79	23.54	0.78
RES	0,5	22,0	9.0	306	41,0	2,73	0	4,41	8,2×10^4^	0,28±0,05	0.61	0.08	14.86	1.01	26.02	1.01
REF	50	17.5	9.0	206	36,0	N.D.	N.D.	2,32	8,1×10^4^	0,08±0,11	0.52	0.28	21.12	0.19	17.98	0.18

*flow cytometry analysis. PO: region influenced by port activity and sewage disposal. RE: open ocean region. S: surface. F: bottom.

The influence of anthropogenic activities was apparent in POS and POF samples as showed by the thermotolerant coliforms results (Table 2). The total cultivable heterotrophic bacterial counts revealed differences between surface and bottom samples at both sites, with higher values determined for POS and POF compared to RES and REF (Table 2). The bacterial abundance measured by flow cytometry also demonstrated higher values for POS and POF compared to RES and REF, with no observed difference between depths (Table 2). Bacterial activity measured by ^3^H-leucine incorporation was higher in superficial waters (POS and RES). Considering the superficial layer, in the sample affected by anthropogenic activity (POS), the bacterial activity was 3.3-fold higher than that in the site influenced by upwelling (RES).

### Microbial diversity and taxonomic composition

**Figure 2:**
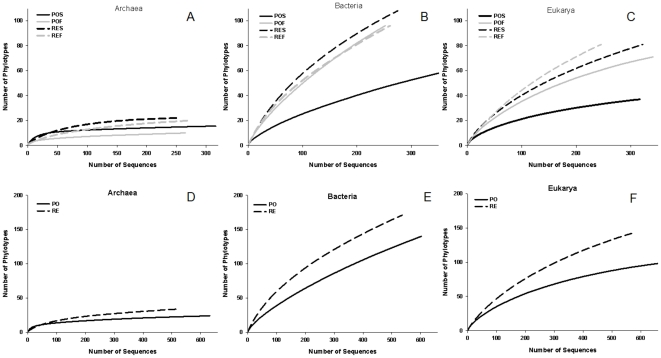
Rarefaction curves calculated using DOTUR_0.03_. It was used the partial sequences of microbial SSU rRNA genes from water samples of Arraial do Cabo region. A, B and C: curves of each library of each domain. D, E and F: curves calculated merging the libraries of the two depths for each point of sample. PO: region influenced by port activity and sewage disposal. RE: open ocean region influenced by upwelling. S: surface. F: bottom (20 m for PO and 50 m for RE).

The estimated values for the OTU richness, diversity index, sample coverage and rarefaction curves of Bacteria, Archaea and Eukarya domains for the four samples analysed are presented in Table 3 and Fig. 2. For all domains, the estimated richness of the phylotypes tended to be lower in the PO samples. The Shannon diversity index was statistically lower in the POS sample than in the other three samples for Bacteria and Eukarya domains (Table 3).

**Table 3:** Estimated OTU richness, diversity indices and estimated sample coverage for SSU rRNA libraries calculated with DOTUR_0.03_.

Library	NS^a^	OTUs^b^	Estimated OTU richness		Shannon	ESC^c^
			ACE	Chao1		
Bacteria						
POS	350	58	163 (108; 278)	132 (89; 236)	2.39 (2.20; 2.57)	0.89
POF	252	96	255 (179; 400)	245 (170; 395)	3.79 (3.61; 3.97)	0.74
PO^d^	602	140	452 (329; 655)	393 (279; 602)	3.41 (3.26; 3.56)	0.98
RES	275	108	217 (171; 296)	191 (151; 270)	4.19 (4.05; 4.32)	0.78
REF	261	96	238 (175; 351)	232 (162; 377)	4.02 (3.87; 4.16)	0.77
RE^e^	536	171	385 (308; 505)	351 (275; 483)	4.47 (4.37; 4.58)	0.82
Eukarya						
POS	317	37	59 (45; 94)	49 (40; 76)	2.30 (2.14; 2.46)	0.95
POF	341	71	105 (87; 144)	93 (80; 124)	3.06 (2.88; 3.24)	0.91
PO^d^	658	98	140 (120; 178)	124 (109; 156)	3.24 (3.12; 3.37)	0.85
RES	322	81	162 (124; 235)	157 (116; 248)	3.40 (3.23; 3.56)	0.86
REF	246	81	184 (136; 275)	158 (118; 242)	3.15 (2.91; 3.40)	0.80
RE^e^	568	142	259 (213; 335)	229 (190; 300)	3.70 (3.54; 3.85)	0.87
Archaea						
POS	366	16	19 (17; 25)	17 (16; 27)	2.19 (2.10; 2.28)	0.99
POF	265	10	16 (11; 45)	16 (11; 48)	1.21 (1.08; 1.34)	0.99
PO^d^	631	24	36 (27; 72)	38 (27; 91)	2.21 (2.12; 2.29)	0.99
RES	251	22	23 (22; 27)	23 (22; 28)	1.72 (1.52; 1.92)	0.99
REF	275	20	25 (21; 41)	24 (21; 42)	1.14 (0.95; 1.34)	0.97
RE^d^	526	34	50 (40; 82)	57 (40; 116)	1.57 (1.41; 1.72)	0.97

a Number of sequences for each library.

b Calculated with DOTUR at the 3% distance level.

c Estimated sample coverage: Cx = 1-(Nx/n), where Nx is the number of unique sequences and n is the total number of sequences [55], [56].

d Calculated by merging the two above libraries.

PO: region influenced by port activity and sewage disposal. RE: open ocean region. S: surface. F: bottom.

**Figure 3:**
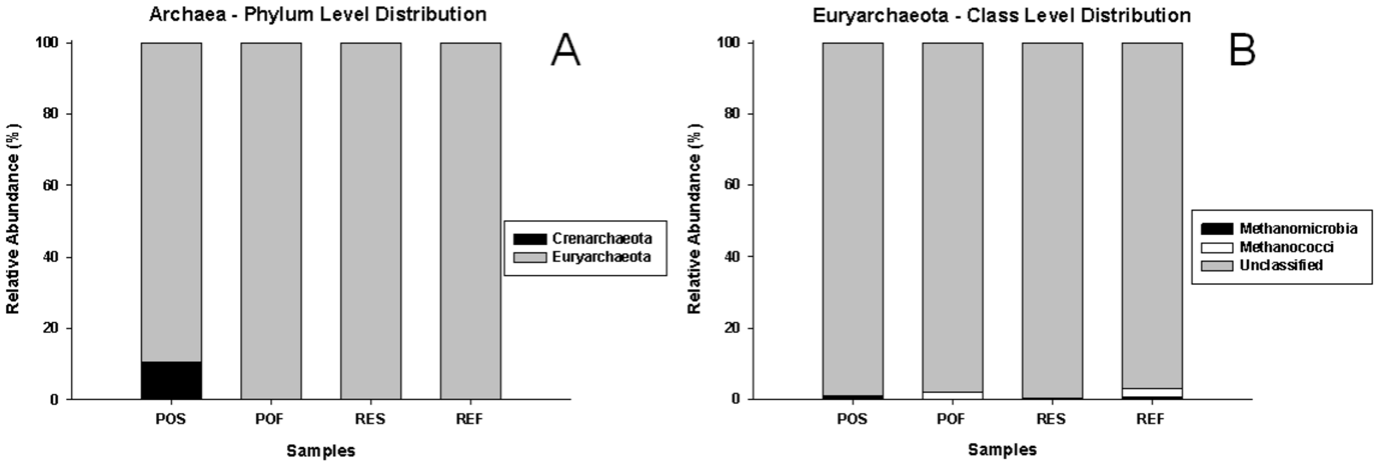
Taxonomic distribution of archaeal 16S rRNA sequences. Classification was performed using the RDP-Classifier (50% of threshold). PO: region influenced by port activity and sewage disposal. RE: open ocean region influenced by upwelling. S: surface. F: bottom (20 m for PO and 50 m for RE).

Unlike the other two domains, the Archaea presented a significantly higher Shannon diversity index associated with the POS sample compared with the other samples (Table 3). The archaeal domain presented the lowest estimated richness and diversity, calculated using DOTUR_0.03_, when compared with the other two domains (Table 3 and Fig. 2). The estimated sample coverage for the Archaea ranged from 97% to 99% (Table 3), suggesting that the majority of the phylotypes of that domain was represented in the generated libraries. The estimated archaeal richness ranged from 16 (POF) to 25 (REF). Despite the lack of statistical differences in the estimated archaeal OTU richness between the samples, the values of the estimated Shannon diversity index presented some significant differences (Table 3). Top water samples presented higher archaeal diversity compared to bottom water samples for each region, and the POS sample contained a higher diversity compared to the other samples. We carried out a comparison using the Fast UniFrac P Test to define the phylogenetic composition similarities between the samples. The results presented in Table 4 (above values) indicate no differences between POS and POF samples (despite the difference in diversity presented in Table 3), whereas all other comparisons presented highly significant differences. These results indicated a possible heterogeneous distribution of the phylotypes between the samples. To investigate the distribution of the phylotypes between the samples based on the taxonomic information, we conducted analyses of classification and phylogeny. According to the RDP-Classifier, the archaeal community of the study area was dominated overwhelmingly by members of the Euryarchaeota phylum because almost all archaeal sequences (96.4%) were classified as Euryarchaeota (Fig. 3a). Surprisingly, the RDP-Classifier was unable to classify the majority of these sequences (99.1%) to the class level, even when a threshold of 50% was used (Fig. 3b). Next, we performed a phylogenetic analysis using a representative sequence of each OTU (total of 54 OTUs) defined by a DOTUR_0.03_ analysis containing all archaeal sequences (1157). After aligning representatives of each OTU using the Aligner tool provided by the SILVA database [61], we found that only uncultivated sequences were recovered as nearest neighbours.

**Table 4:** Statistic significance^1^ (*P* values) of differences between prokaryotic communities of water samples of Arraial do Cabo region calculated based on partial sequences of 16S rRNA.

		POS	POF	RES	REF			POS	POF	RES	REF
		Corrected 2						Not Corrected			
			Archaea						Archaea		
**POS**		-	0.684	<0.001	<0.001	**POS**		-	0.101	<0.001	<0.001
**POF**	Bacteria	1	-	<0.001	<0.001	**POF**	Bacteria	0.444	-	<0.001	<0.001
**RES**		0.762	0.99	-	<0.001	**RES**		0.128	0.137	-	<0.001
**REF**		0.42	1	0.168	-	**REF**		0.071	0.301	0.023	-

1 *P* values of UniFrac P Test are calculated based on 1,000 permutations (pairwise differences). (<0.001) highly significant; (0.001–0.01) significant; (0.01–0.05) marginally significant; (0.05–0.1) suggestive; (>0.1) not significant.

2 *P* values corrected for multiple comparisons using the Bonferroni correction.

PO: region influenced by port activity and sewage disposal. RE: open ocean region. S: surface. F: bottom.

Unlike the RDP-Classifier classification (which classified most of the sequences as Euryarchaeota), most of the nearest neighbours were defined as crenarchaeota-like (uncultivated) in GenBank. We then added to the data set a representative sequence of each subgroup of Archaea-type strains defined by Yarza and colleagues [63]. A phylogenetic analysis was then performed, and the generated phylogram is shown in Fig. S1. The type strain sequences were grouped into two clades named Euryarchaeota and Crenarchaeota (Fig. S1). Seven and forty-seven of the generated clones were grouped in the Euryarchaeota and Crenarchaeota clades, respectively, indicating that the most of generated sequences (95%) belonged to archaeal groups that have not yet been characterised.

The Euryarchaeota clade was clearly separated into two clades: I and II. Clade I contained only type strain sequences, and the generated sequences were grouped into clade II. Even within this clade, the generated sequences appeared to be more related to uncultivated Euryarchaeota sequences distributed in the three subclades (II.a, II.b and II.c, Fig. S1). In the same manner, the 47 sequences belonging to the Crenarchaeota clade (grouped in seven OTUs) appeared to be more related to uncultivated Crenarchaeota sequences than to the type strains (Fig. S1). OTUs 19, 22 and 25 were classified as Crenarchaeota by the RDP-Classifier, which is in agreement with the phylogram. However, three other OTUs (numbers 5, 10 and 15) were classified as unclassified Euryarchaeota despite their positions in the phylogram. Nonetheless, OTU 24 was classified as unclassified Archaea. Most of the generated sequences (70.2%) were grouped in clade IV (Fig. S1) and showed a wide distribution in the four samples.

Excluding the low Shannon index values presented by POS, as mentioned above, and the significantly higher diversity of RES samples when compared with POF samples, the estimated richness and diversity of bacterial phylotypes presented no statistical differences between the samples (Table 3). No statistical differences were detected using the Fast UniFrac P Test (Table 4 – below values), which revealed that the bacterial communities of the samples were similar.

**Figure 4:**
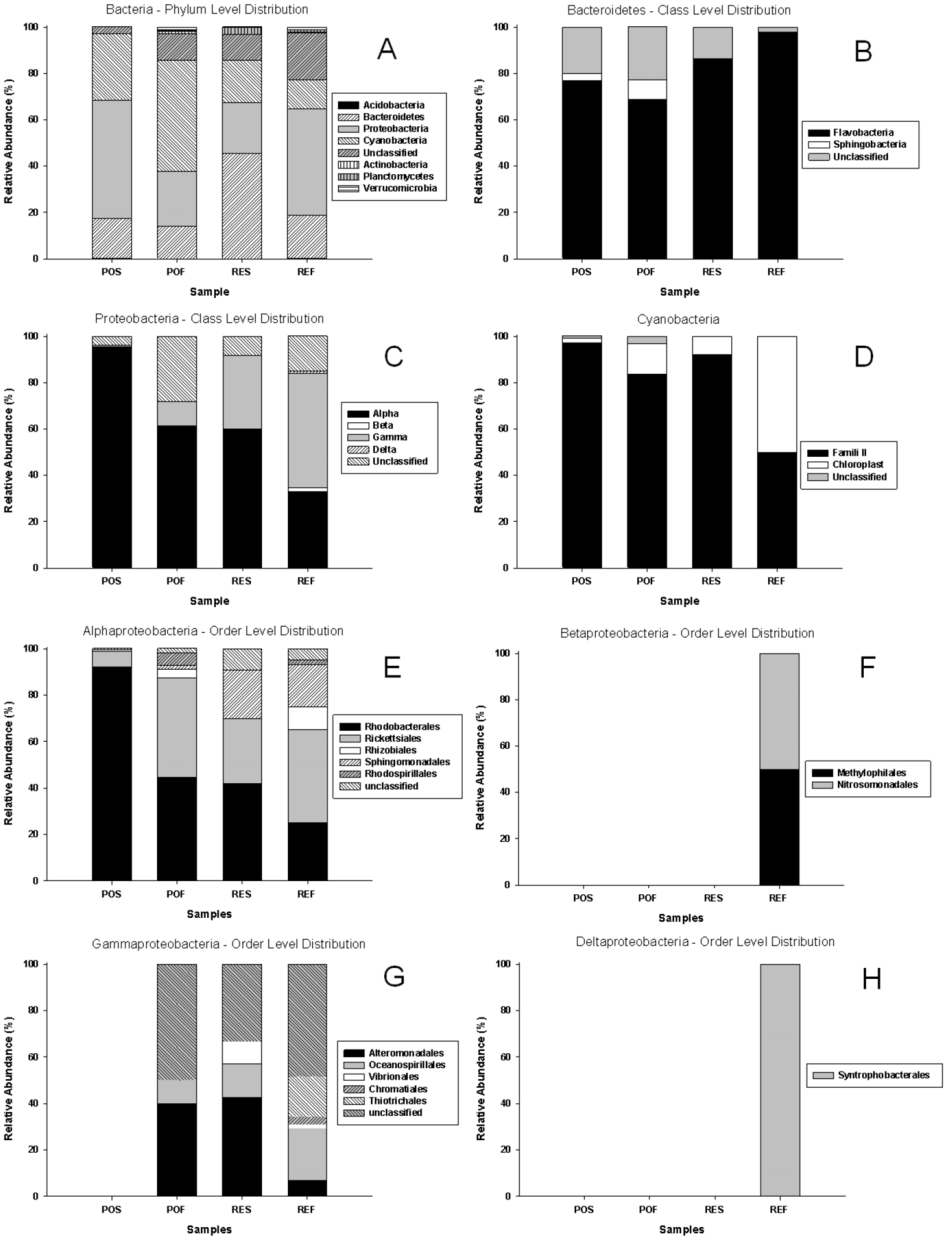
Taxonomic distribution of bacterial 16S rRNA sequences. Classification was performed using the RDP-Classifier (50% of threshold). PO: region influenced by port activity and sewage disposal. RE: open ocean region influenced by upwelling. S: surface. F: bottom (20 m for PO and 50 m for RE).

Figure 4a shows the relative abundance of the phyla detected in the four samples by RDP-Classifier analysis. The communities were strongly dominated by members of the Bacteroidetes, Proteobacteria and Cyanobacteria. Bacteroidetes was the most abundant phylum in RES, demonstrating an intermediate occurrence in the other three samples (Fig. 4a). Most of the Bacteroidetes sequences found in all samples belonged to the Flavobacteria class, whereas members of the Sphingobacteria class were detected only in POS and POF samples (Fig. 4b).

The Proteobacteria was the most abundant phylum in POS and REF samples (Fig. 4a). Most of the Proteobacteria sequences belonged to the Alphaproteobacteria class, except for the REF sample, which was dominated by the Gammaproteobacteria class (Fig. 4c). Sequences of Beta- and Deltaproteobacteria were found only in the REF sample (Fig. 4c).

Cyanobacteria were the most abundant phylum in the POF sample but were detected in all samples in considerable amounts (Fig. 4a). For POS, POF and RES, most of the Cyanobacteria sequences belonged to Family II according to the RDP-Classifier classification, and for the REF sample, 50% of the sequences classified as Cyanobacteria-like represented chloroplast sequences (Fig. 4d).

**Figure 5:**
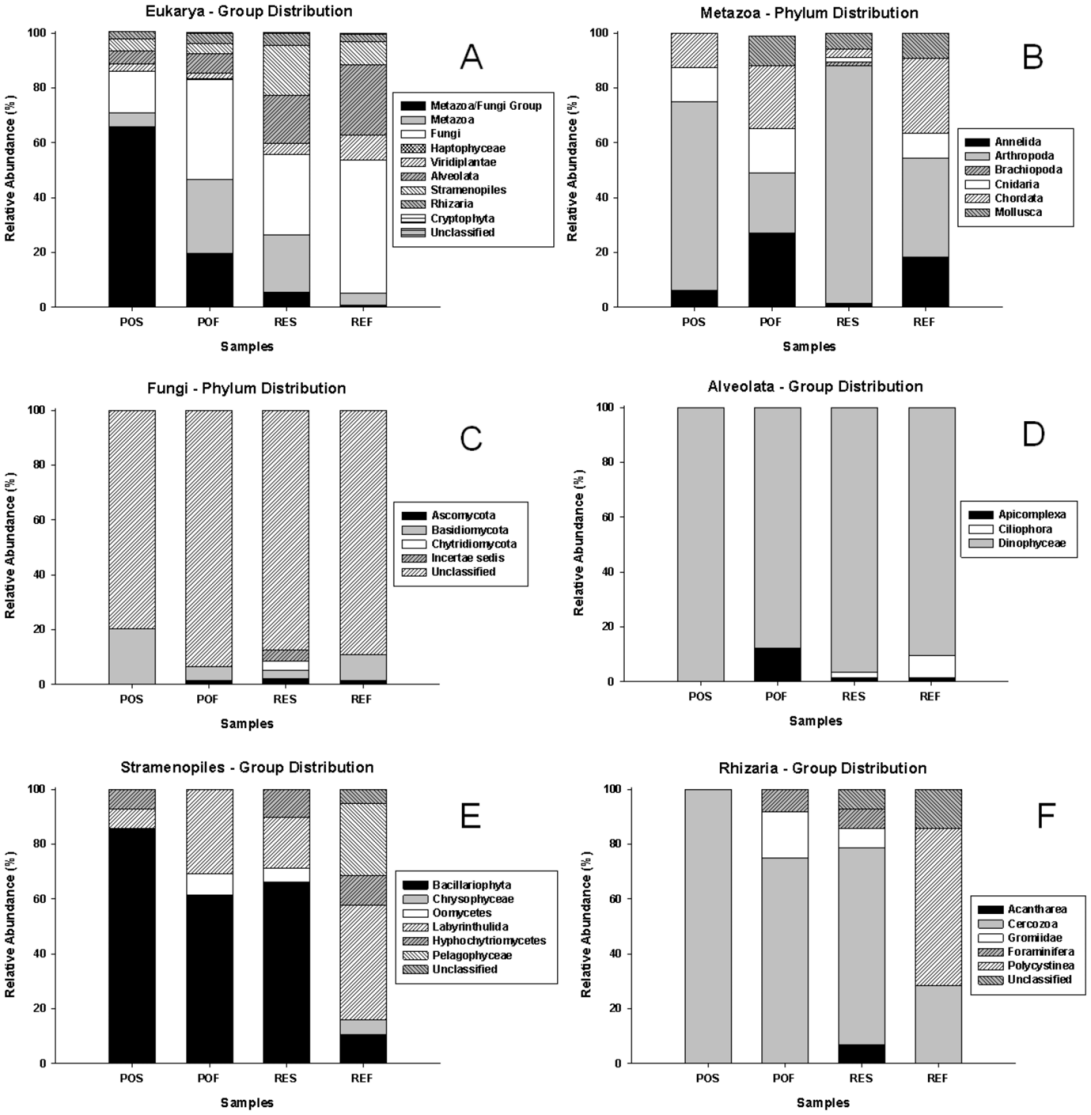
Taxonomic distribution of 18S rRNA sequences. Affiliation was performed using NCBI-Blast searches. PO: region influenced by port activity and sewage disposal. RE: open ocean region influenced by upwelling. S: surface. F: bottom (20 m for PO and 50 m for RE).

We used the NCBI-BLAST search and evaluated the classification of NCBI Taxonomy to sort the generated sequences using specific 18S rRNA primers. Table S1 shows the output occurrences of the most similar sequences and the most similar sequences of a characterised organism deposited in GenBank using a representative sequence of each OTU defined by DOTUR_0.03_ as input data. Although previous studies have demonstrated that cloning approaches using a single pair of primers are not sufficient to cover the overall environmental diversity of microeukaryotes [64]–[69], we decided to describe the partial diversity detected, considering the lack of diversity information for the study area and other Brazilian marine environments using molecular approach. Moreover, a comparative analysis can be generated considering the use of the same approach for the four samples. In general, we note that small (<97%) similarity values are frequently encountered in BLAST searches (Table S1), indicating that the Arraial do Cabo region comprises uncharacterised microeukaryotes. We found that the sequences were associated with nine major groups (Fig. 5a).

The POS sample was dominated by sequences of an Incertae sedis of the Metazoa/Fungi major group, and most of these sequences match with sequences of the order Ichthyosphonida, with similarity values ranging from 88 to 95% (Fig. 5a and Table S1). The Metazoa was the second most abundant group in the POF and RES samples. Within the Metazoa group, we discovered sequences that align with sequences of organisms affiliated with six phyla (Fig. 5b). The Arthropoda presented a high relative abundance of Metazoa in POS, RES and REF samples, whereas the POF sample presented a more uniform distribution of sequences between the phyla (Fig. 5b). The Fungi group showed a high relative abundance in the POF, RES and REF samples (Fig. 5a). However, most sequences could not be classified in subsequent taxonomic levels (Fig. 5c). The other groups, which were detected in lower relative abundance than that classified as Fungal sp., were affiliated with the phyla Ascomycota, Basidiomycota and Chytridiomycota (Fig. 5c). The Alveolata group presented a greater relative abundance in RES and REF samples compared with POS and POF (Fig. 5a). Most of the sequences classified as Alveolata were members of the class Dinophyceae (dinoflagellates), whereas the other sequences were members of the phylum Apicomplexa and the group Ciliophora (ciliates) (Fig. 5d). The Stramenopiles group demonstrated a higher relative abundance in RES sample compared with POS, POF and REF samples (Fig. 5a). Within Stramenopiles group, members of the phylum Bacillariophyta presented the higher relative abundance in POS, POF and RES samples, whereas members of the order Labyrinthulida showed the higher relative abundance compared to the other phyla in REF sample (Fig. 5e).

### PCR-DGGE profiles

**Figure 6:**
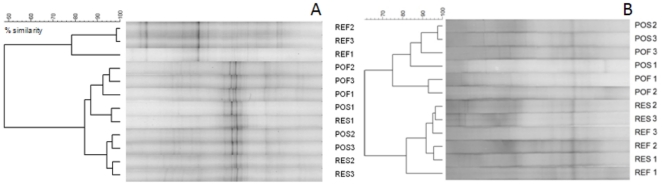
DGGE profiles of PCR-amplified SSU rRNA gene fragments of Bacteria and Eukarya. Triplicate samples are used. Clustering analysis was based on Pearson's correlation index and the unweighted pair-group method with arithmetic averages. PO: region influenced by port activity and sewage disposal. RE: open ocean region influenced by upwelling. S: surface. F: bottom (20 m for PO and 50 m for RE).

To obtain an overview of the bacterial and eukaryotic community similarity, we performed DGGE analyses using partial sequences of SSU rRNA. When the 16S rRNA of Bacteria was analysed, the result revealed two main clusters (Fig. 6a): one group of REF samples and the other of the other three sample types. A simple view of the gel indicated that the communities comprising the larger cluster were more similar to each other than to any REF repetition. Within the larger cluster, we observed the formation of two subclusters, one containing the POF repetition cluster and the other containing a POS and a RES repetition. Within the other subcluster, the remaining POS and RES repetitions grouped together. When the 18S rRNA of Eukarya was analysed, the result revealed two main clusters (Fig. 6b): one group of samples of the area influenced by anthropogenic activity (POS and POF) and the other of samples of the area influenced by upwelling (RES and REF). Within each main cluster, repetitions of sampling did not show tendency to group together, indicating a less influence of depth related to the definition of the eukaryotic assemblages (Fig. 6b).

## Discussion

### Bacterial counting and production

Bacterial counting and production were higher in the top samples and in the samples influenced by anthropogenic activities (Table 2). Normally, low temperature decreases bacterial production [70], [71], and Guenther and colleagues [20] suggested a great variability in the plankton interactions during a downwelling-upwelling cycle. During upwelling events, bacterial production decreased from 0.1 to 0.02 µC.L^−1^.h^−1^, and superficial waters presented higher bacterial production than samples collected at 40 m depth. Carvalho & Gonzalez-Rodriguez [38] simulated mixtures of Arraial do Cabo water types and observed that bacterial production induced plankton primary activity but was benefited after maximum phytoplankton production rates. The dynamics observed in total production, growth and biomass turnover rates should consider the complex shifts in the distribution of activity and physiological states within the community assemblage [72].

### Microbial diversity and assemblages

We found a low number of phylotypes of Archaea (Table 3) in comparison with previous reports that investigated other environments like soils [73] or sediments [74] using the same approach. However, richness ranging from 16 to 25 can represent higher diversity in Arraial do Cabo when compared with other marine environments. For example, Galand and colleagues [75] found a richness (estimated by Chao1) that ranged from 3 to 7 phylotypes in arctic marine deep waters (62 to 180 m).

The results confirmed the tendency of crenarchaeotal groups to dominate marine environments (Figs. 3 and S1), especially the marine group I crenarchaeotes (MGI). According to DeLong [76], MGI is a widespread archaeal group that appears to be derived from thermophilic ancestors that invaded diverse non-extreme environments. According to the author, MGI and Group II Euryarchaeota (MGII) are the two most abundant archaeal planktonic groups. Some studies have demonstrated that MGI often represents a substantial fraction of the picoplanktonic community [76], [77]. According to Karner and colleagues [78], there are approximately 1.3×10^28^ archaeal cells in the world's oceans, of which ∼20% are Crenarchaeota. Despite the evidence for a co-dominance of MGI and MGII in marine environments [76], [79], the distribution of OTUs and nearest neighbours in the phylogram (Fig. S1), BLAST searches using the generated clones and nearest neighbours (data not shown) and the finding that only seven generated sequences formed a clade containing a MGII member (Clade II.C – Fig. S1) led us to believe that MGI is the most abundant archaeon in the waters of the Arraial do Cabo region.

Some authors have demonstrated a shift of the MGI/MGII dominance according to the season in which the samples were collected [79], [80]. Thus, we must consider the seasonal variance of this MGI dominance.

The anthropogenic impact of sewage disposal near to PO site should be favourable for Crenarchaeota, once this group use ammonia as its sole source of energy [81]. However, the significantly higher abundance of Crenarchaeota than Euryarchaeota was also observed in RE samples. These results suggested that the ammonification process could be associated to both natural (upwelling) and artificial (sewage) eutrophication occurring in the Arraial do Cabo region. According to Kuypers and colleagues [1], 50% of marine N loss is associated to upwelling areas and associated oxygen minimum zones (OMZ). Lam and colleagues [82] showed that MGI plays an important role in the nitrification steps of this loss.

We found an unexpectedly high relative abundance of member of Fungi group in POF, RES and REF samples (Fig. 5a) within the eukaryotic assemblage represented by 18S rRNA sequences. However, most sequences could not be classified in subsequent taxonomic levels (Fig. 5c) because the output of the BLAST search indicated an unclassified fungus (Fungal sp., access number GQ120167) as the sequence related (88% to 100% similarity, Table S1) to these widespread sequences found in the study area. GQ120167 is a complete 18S rRNA gene sequence of an uncharacterised fungus isolated from anoxic water of the Arabian Sea [65]. According to another study by the same group, fungal isolates obtained from anoxic waters of the Arabian Sea can play an important role in the N cycle in this environment, participating in denitrification [83].

### Spatial zonation of microbial phylotypes

Some differences related to the distribution of the microbial populations could even be detected between samples that did not display differences related to α-diversity or phylogenetic (Fast Unifrac) analysis.

The differential spatial distribution of phylotypes was clearly apparent when we analysed the disposal of the archaeal sequences in a phylogram (Fig. S1), in which some clusters were formed exclusively by sequences belonging to a sample collection point. This was the case for clusters VII.b and XI, which contained only RE sequences, and cluster VIII, which contained only PO sequences.

Despite the lack of significant differences between the bacterial community compositions of the samples when we analysed the core data using Fast Unifrac, the bacterial communities presented some specificity in the sequence distribution between samples. This was the case for the Planctomycetes phylum, which was visible only in the upwelling influenced site (Fig. 4a). We discovered a total of twelve clones of the Planctomycetes, eleven of which were found in RE samples. All sequences were classified as members of *Planctomycetales* order by the RDP-Classifier: one clone was found in the POF sample, three clones in the REF sample and eight clones in the RES sample. These clones were represented by OTUs that formed a separate clade in the phylogram (Fig. S2). Together with archaeon MGI nitrifying organisms [83], groups of *Planctomycetales* are responsible for the anaerobic oxidation of ammonium (anammox bacteria) in suboxic zones [1], [13]. According to Kuypers and colleagues [1], ∼1% of the cells presented in the water of the Benguela upwelling system were related to anammox bacteria. The authors found a depletion of ammonium followed by increases in nitrite and nitrate concentrations at depths of 30–80 m. These concentrations are in agreement with the concentrations determined in our study, and a depletion of ammonium followed by increases in nitrite and nitrate concentrations was detected in the REF sample (50 m sample) when compared with the other three samples (Table 2). However, we did not find suboxic conditions in the studied area (Table 2). As showed also by previous studies, we hypothesise that the microorganisms potentially involved in the utilisation of fixed inorganic N (e.g., MGI, *Planctomycetales*, uncharacterised fungi) are facultative anaerobic organisms.

The Planctomycete sequences obtained in our study were affiliated with members of the genus *Rhodopirellula* (RDP-Classifier threshold 100% - data not shown). The members of *Rhodopirellula* are considered aerobic heterotrophic bacteria [84]. However, sequencing of the genome of *Rhodopirellula baltica* [85] revealed the presence of genes related to fermentation. Furthermore, it is known that some Planctomycete strains are considered microaerobic, oligotrophic, heterotrophic microorganisms and that the genus *Pirellula* includes facultative nitrate reducers [84], [86]. One of the nearest neighbours that clustered with the Planctomycetes clade in the phylogram shown in Fig. S2 belonged to a strain of *Pirellula* sp. According to Woebken and colleagues [13], even under water-oxic conditions, anammox bacteria (e.g., *Planctomycetales*) can oxidise ammonium when they are associated with the interior of the suspended particles, where the oxygen is depleted by groups of heterotrophic bacteria.

The zonation of Eukarya domain was pronounced to the order Ichthyophonida (296 sequences) that belong to an Incertae sedis of the Metazoa/Fungi main group (Fig. S3). Approximately 93% of these sequences were found in the samples that were influenced by anthropogenic activity, especially in the top sample (∼72%), whereas only ∼7% of the sequences were found in samples that were influenced by upwelling (Table S1).

Most of these sequences were related to an Ichthyophonida organism isolated from marine invertebrate digestive tracts (access no. EU124916) [87] and to *Ichthyophorus hoferi* (EU332789), a well-known cosmopolitan parasite of marine fishes [88]. However, the similarity between these two sequences and our clones was low, ranging from 88% to 95% (Table S1). Our sequences were more similar (93% to 99%) to two other environmental sequences: AY884988 [89], found in a Mid-Atlantic estuary, and AY331730 [90], found in the surface water of the Bay of Fundy (East coast of North America). This finding led us to believe that the generated sequences belonged to uncharacterised microeukaryotes.

### Natural and anthropogenic impact

The distribution of some bacterial groups between REF and the other sites was clearly in the present studied area (Fig. 6a; Table 4). Traditionally, depth is accepted as the most important factor defining the zonation of prokaryotic occurrence in marine water [42], [77], [78], [82], [91]–[94]. It is well established that vertical zonations can be determined by gradients of light intensity, temperature, hydrostatic pressure, nutrient availability, oxygen content, among others. Superficial water contains more bacterial activity due to the presence of primary producers, whereas in deep waters, the prokaryotes should demonstrate a greater association with particles. Although most prokaryotes are free-living in oceanic water columns, the detachment of cells associated with sinking particles is an important mechanism that supplies free-living prokaryotic cells in deeper waters [95].

The distribution of microorganisms could also be defined by the characteristics of the different water masses [96]–[98]. Observing the low temperature reported in REF (table 2), SACW was presented at the deep waters from the upwelling influenced area. The different water conditions could explain the formation of different clusters of archaea-containing phylotypes exclusively from RES/REF (upwelling influenced area) or POS/POF (anthropogenic influenced area) samples (Fig. S1), as well as for the Eukarya domain (Fig. 6b). Moreover, the DGGE dendrogram do not show the formation of subclusters contrasting superficial (S) and deep (F) samples. We concluded that the temperature determined by the water type should be the main regulating factor for microbial assemblages and that the anthropogenic impact decreases its diversity in the Arraial do Cabo upwelling area.

## Supporting Information

Figure S1 **Phylogram of the archaeal 16S rRNA phylotypes.** A representative sequence of each OTU determined by DOTUR_0.03_ and the nearest neighbors obtained by using of the aligner tool of the silva database project were used. The sequences of type strains used in the phylogram were obtained from the All-Species Living Tree project (Yarza *et al*., 2008). The number and the letters after the OTU identification indicate the number of sequences clustered in each OTU and the sample where the sequences were found, respectively. PO – area influenced by anthropogenic activity; RE – open ocean area influenced by the upwelling phenomena; S – superficial sampling; F – bottom sampling (20 m for PO and 50 m for RE). The phylogram was calculated with MEGA 4.0 using neighbor-joining method and Jukes-Cantor model. Numbers at the branches show bootstrap percentages (above 50% only) after 1000 replications of bootstrap sampling. The sequence of *Abiotrophia defectiva* (Bacteria) was set as outgroup.
(TIF)

Figure S2 **Phylogram of the bacterial 16S rRNA phylotypes.** A representative sequence of each OTU determined by DOTUR_0.03_ and the nearest neighbors obtained by using of the aligner tool of the silva database project were used. The number and the letters after the OTU identification indicate the number of sequences clustered in each OTU and the sample where the sequences were found, respectively. PO – area influenced by anthropogenic activity; RE – open ocean area influenced by the upwelling phenomena; S – superficial sampling; F – bottom sampling (20 m for PO and 50 m for RE). The phylogram was calculated with MEGA 4.0 using neighbor-joining method and Jukes-Cantor model. Numbers at the branches show bootstrap percentages (above 50% only) after 1000 replications of bootstrap sampling. The sequence of *Caldisphaera draconis* (Archaea) was set as outgroup.
(TIF)

Figure S3 **Phylogram of the 18S rRNA phylotypes.** A representative sequence of each OTU determined by DOTUR_0.03_ and the nearest neighbors obtained by using of the aligner tool of the silva database project were used. The number and the letters after the OTU identification indicate the number of sequences clustered in each OTU and the sample where the sequences were found, respectively. PO – area influenced by anthropogenic activity; RE – open ocean area influenced by the upwelling phenomena; S – superficial sampling; F – bottom sampling (20 m for PO and 50 m for RE). The phylogram was calculated with MEGA 4.0 using neighbor-joining method and Jukes-Cantor model. Numbers at the branches show bootstrap percentages (above 50% only) after 1000 replications of bootstrap sampling.
(TIF)

Table S1 **Closest relative sequences obtained with NCBI-Blast search using generated partial 18S rRNA sequences from water samples of Arraial do Cabo region.** PO: region influenced by port activity and sewage disposal. RE: open ocean region. S: surface. F: bottom.
(PDF)
