# Supplementary figures and images for: Microbial Diversity of a Brazilian Coastal Region Influenced by an Upwelling System and Anthropogenic Activity
Source: PLoS One. 2011 Jan 27;6(1):e16553. doi: 10.1371/journal.pone.0016553 (PMC3029357; doi:10.1371/journal.pone.0016553)

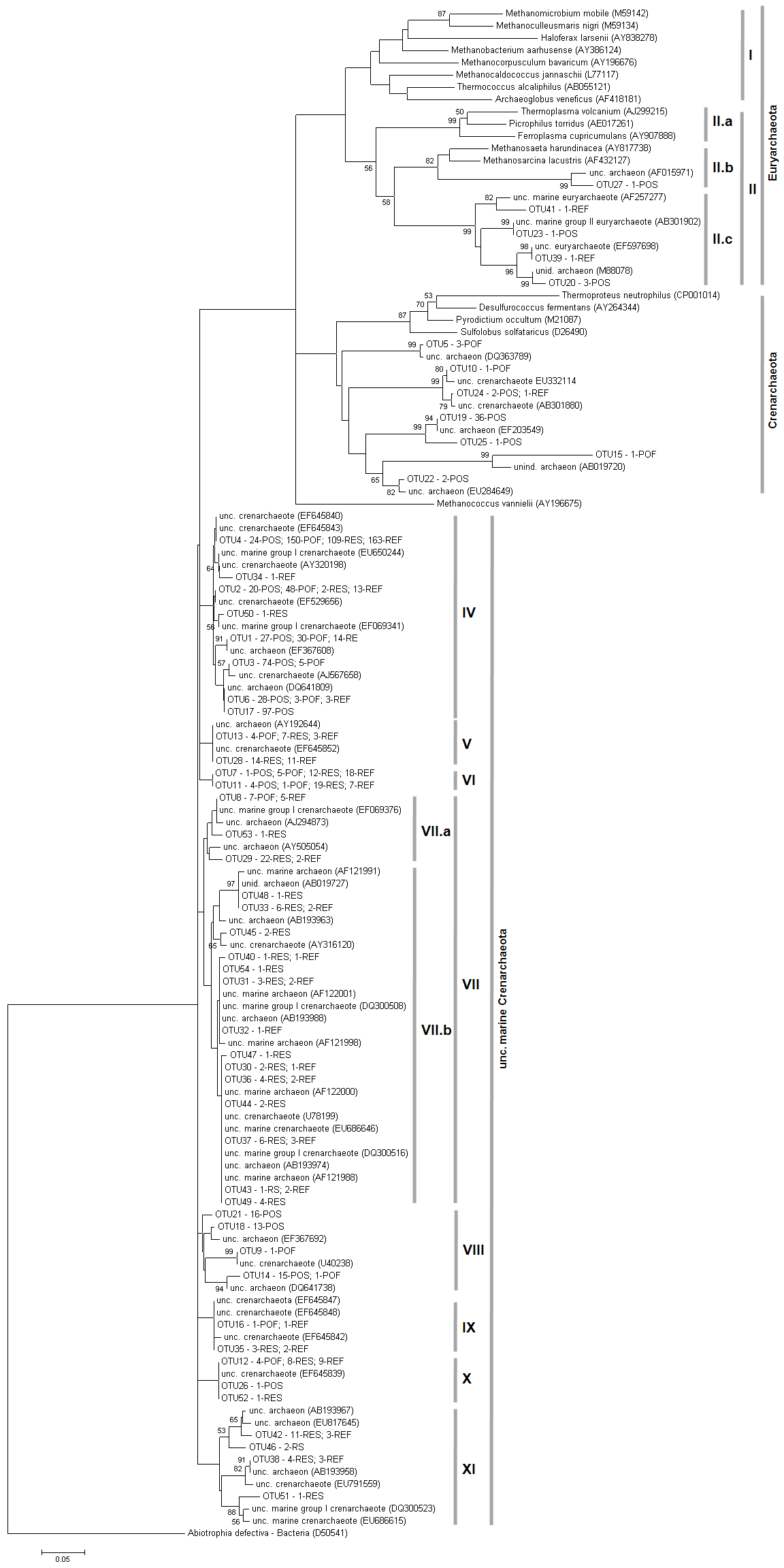

Supplement: Figure S1 — Phylogram of the archaeal 16S rRNA phylotypes. A representative sequence of each OTU determined by DOTUR0.03 and the nearest neighbors obtained by using of the aligner tool of the silva database project were used. The sequences of type strains used in the phylogram were obtained from the All-Species Living Tree project (Yarza et al., 2008). The number and the letters after the OTU identification indicate the number of sequences clustered in each OTU and the sample where the sequences were found, respectively. PO – area influenced by anthropogenic activity; RE – open ocean area influenced by the upwelling phenomena; S – superficial sampling; F – bottom sampling (20 m for PO and 50 m for RE). The phylogram was calculated with MEGA 4.0 using neighbor-joining method and Jukes-Cantor model. Numbers at the branches show bootstrap percentages (above 50% only) after 1000 replications of bootstrap sampling. The sequence of Abiotrophia defectiva (Bacteria) was set as outgroup. (TIF) [file pone.0016553.s001.tif]

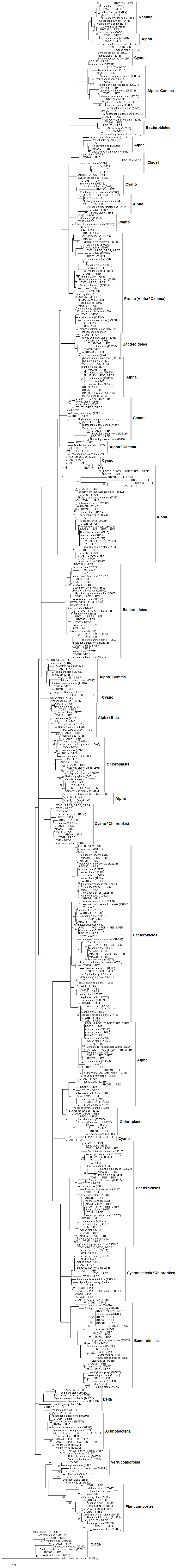

Supplement: Figure S2 — Phylogram of the bacterial 16S rRNA phylotypes. A representative sequence of each OTU determined by DOTUR0.03 and the nearest neighbors obtained by using of the aligner tool of the silva database project were used. The number and the letters after the OTU identification indicate the number of sequences clustered in each OTU and the sample where the sequences were found, respectively. PO – area influenced by anthropogenic activity; RE – open ocean area influenced by the upwelling phenomena; S – superficial sampling; F – bottom sampling (20 m for PO and 50 m for RE). The phylogram was calculated with MEGA 4.0 using neighbor-joining method and Jukes-Cantor model. Numbers at the branches show bootstrap percentages (above 50% only) after 1000 replications of bootstrap sampling. The sequence of Caldisphaera draconis (Archaea) was set as outgroup. (TIF) [file pone.0016553.s002.tif]

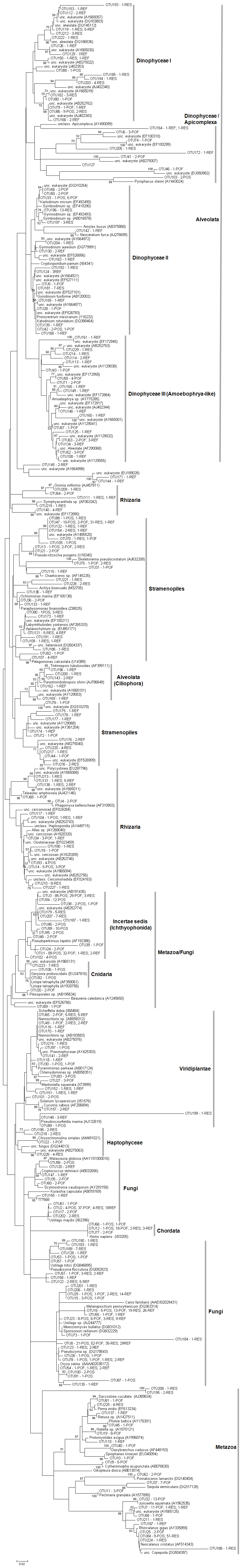

Supplement: Figure S3 — Phylogram of the 18S rRNA phylotypes. A representative sequence of each OTU determined by DOTUR0.03 and the nearest neighbors obtained by using of the aligner tool of the silva database project were used. The number and the letters after the OTU identification indicate the number of sequences clustered in each OTU and the sample where the sequences were found, respectively. PO – area influenced by anthropogenic activity; RE – open ocean area influenced by the upwelling phenomena; S – superficial sampling; F – bottom sampling (20 m for PO and 50 m for RE). The phylogram was calculated with MEGA 4.0 using neighbor-joining method and Jukes-Cantor model. Numbers at the branches show bootstrap percentages (above 50% only) after 1000 replications of bootstrap sampling. (TIF) [file pone.0016553.s003.tif]
