# Supplementary material for: Microbial Diversity of a Brazilian Coastal Region Influenced by an Upwelling System and Anthropogenic Activity
Source: PLoS One. 2011 Jan 27;6(1):e16553. doi: 10.1371/journal.pone.0016553 (PMC3029357; doi:10.1371/journal.pone.0016553)
Supplement: Table S1 — Closest relative sequences obtained with NCBI-Blast search using generated partial 18S rRNA sequences from water samples of Arraial do Cabo region. PO: region influenced by port activity and sewage disposal. RE: open ocean region. S: surface. F: bottom. (PDF) [file pone.0016553.s004.pdf]

Table S1 Closest relative sequences obtained with NCBI-Blast search using generated partial 18S rRNA sequences from water samples of Arraial do Cabo region. PO: region influenced by port activity and sewage disposal. RE: open ocean region. S: surface. F: bottom

**Phylogenetic affiliation**	**Closest described relative**		**Closest phylotype**					
	**Species or strain (accession no.)**	**Sim (%)**	**Sequence (accession no.)**	**Sim (%)**	**POS**	**POF**	**RES**	**REF**
					No. of clones			
Metazoa/Fungi group								
Incertae sedis	*Amoebidium parasiticum* Y19155	91	Unc. Eukaryote DQ234281	94	2			
	Ichthyophonida sp. EU124916	90-95	Unc. Eukaryote AY884988	93-99	103	35	1	2
	Ichthyophonus hoferi EU332789	88-91	Unc. Eukaryote AY884988 / AY331730	96-98	104	32	3	-
	Pseudoperkinsus tapetis AF192386	86-89	Unc. Eukaryote DQ234281 / FJ221394	95-96	-	-	14	-
Metazoa								
Annelida	*Anaitides* sp. AY894293	94	*Anaitides* sp. AY894293	94				2
	Chrysoxys sp. AF123302	97	Unc. Stramenopile EU143916	97		2		
	Dasybranchus caducus AF448153	98	*Dasybranchus caducus* AF448153	98		1		
	Hydroides norvegica AY611452	92	*Hydroides norvegica* AY611452	92		7		
	Karlodinium micrum EF492490	98	*Karlodinium micrum* EF492490	98	1	5		
	Karlodinium micrum EF492490	83	Unc. Eukaryote AJ402340	89			1	
	Lecudina polymorpha AY196707	88	Unc. Eukaryote EU050982	92		1		
	Pectinaria gouldii DQ790091	96	*Pectinaria gouldii* DQ790091	96		3		
	Pholoe baltica AY176301	99	*Pholoe baltica* AY176301	99		1		
	Pomatoceros lamarckii DQ140404	98	*Pomatoceros lamarckii* DQ140404	98		2		
	Selenidium serpulae DQ683562	93	*Selenidium serpulae* DQ683562	93		2		
	Spiophanes kroeyeri EU340096	98	*Spiophanes kroeyeri* EU340096	98		1		
Arthropoda	*Clausocalanus ingens* AF367718	97-99	*Clausocalanus ingens* AF367718	97-99	9		51	3
	Ctenocalanus vanus AF462320	91-99	*Ctenocalanus vanus* AF462320	91-99			4	
	Cytheromorpha acupunctata AB076630	95	*Cytheromorpha acupunctata* AB076630	95		5		
	Ichthybotus hudsoni AY749892	75	*Ichthybotus hudsoni* AY749892	75			2	
	Neocalanus cristatus AF514344	93-96	Unc. Eukaryote AY665127 / AY665127	96-99		4		
	Paracyclopina nana FJ214952	96	Unc. Ekaryote DQ344714	98		11	1	1
	Phthirus pubis AF139485	99	Unc. Pucciniomycotina EU647044	99	2			
Brachiopoda	*Hemithiris psittaceae* U08322	76	*Hemithiris psittaceae* U08322	76			1	
Cnidaria	*Clytia hummelincki* AY789745	88	Unc. Eukaryote EU189028	94				1
	Cordagalma cordiforme AY937317	99	Unc. Eukaryote AY665131	99			1	
	*Geryonia proboscidalisv*EU247816	99	*Geryonia proboscidalisv*EU247816	99	1			
	Junceella aquamata AY962535	99	*Junceella aquamata* AY962535	99		13		
	Liriope tetraphylla AY920756	94-95	*Liriope tetraphylla* AY920756	94-95	1	2		

Chordata	Human DNA sequence CT476837	96-99	Human DNA sequence CT476837	96-99	2	21	2	3
Mollusca	*Hiatella arctica* AM774511	99	*Hiatella arctica* AM774511	99		9		
	Ostrea edulis U88709	99	Ostrea edulis 18S U88709	99		1		
	Perna canaliculus DQ640523	100	*Perna canaliculus* DQ640523	100			4	
	Pyrunculus sp. DQ923465	97	*Pyrunculus* sp. DQ923465	97				1
Choanoflagellida	*Stephanoeca diplocostata* AF084235	90	Unc. Eukaryote EF526904	90		1		
Fungi								
Ascomycota	*Dothioraceae* sp. EF060795	99	Unc. Ascomycete EU409872	98				1
	Pneumocystis carinii X12708	91	*Pneumocystis carinii* X12708	91				1
	Pleosporales sp. EU594362	93	*Pleosporales* sp. EU594362	93		2		
	Taphrina johansonii AJ495835	88	*Taphrina johansonii* AJ495835	88			2	
Basidiomycota	*Cryptococcus vishniacii* EU723509	100	Unc. Agaricomycotina EU647139	100				1
	Sporisorium sp FJ517760	92	Unc. Eukaryote EU189028	96				1
	Ustilago maydis X62396	97	*Ustilago maydis* X62396	97	8	6	3	9
	Ustilago sp. AJ244777	96	*Ustilago* sp. AJ244777	96	2			
Chytridiomycota	*Chytriomyces hyalinus* DQ536487	88	Unc. Eukaryote EU091849	88			3	
Incertae sedis	*Basidiobolus haptosporus* AF368504	93	Unc. Eukaryote EU050973	98			4	
Unclass. Fungi	Fungal sp. GQ120167	88-100	Fungal sp. GQ120167	88-100	39	117	83	106
	Fungal sp. GQ120167	92	Unc. Eukaryote EU189028	96				1
Haptophyceae								
Prymnesiales	*Chrysochromulina* sp DQ980478	99	*Chrysochromulina* sp DQ980478	99		1		
Viridiplantae								
Chlorophyta	*Chlamydomonas* sp. AF514399	96	Unc. Eukaryote AB238085	96	3			
	Chlorella sp. AB437244	95	*Chlorella* sp. AB437244	95				2
	Coccomyxa parasitica EU127469	95	Coccomyxa parasitica EU127469	95				2
	Mantoniella squamata X73999	95	*Mantoniella squamata* X73999	95		3		
	Nannochloris sp. AB183585	96-100	Unc. Eukaryote FJ221502 / AB275076	96-100		3	7	8
	Ostreococcus lucimarinus CP000592	98	*Ostreococcus lucimarinus* CP000592	98			1	1
	Ostreococcus lucimarinus CP000592	87	Unc. Eukaryote AF525861	87			1	1
	Picochlorum sp. AB488603	99	Unc. Eukaryote AB275076	99				1
	Prasinophyta sp. AY425302	99	Unc. Prasinophyte AY425303	99			1	
	Pycnococcus provasolii AY425305	100	*Pycnococcus provasolii* AY425305	100				3
	Pycnococcus sp. AB058359	89	*Pycnococcus* sp. AB058359	89	1			
	Pyramimonas aureus AB052289	99	*Pyramimonas aureus* AB052289	99	1	1		
	Tetraselmis sp. AY425299	97	*Tetraselmis* sp. AY425299	97	1			
Streptophyta	*Luffa quinquefida* AF008957	99	Unc. Eukaryote FJ785978	99				2
	Mesotaenium chlamydosporum AJ553923	84	Unc. Eukaryote AY665068	96			1	
	Netrium interruptum AJ428071	91	Environmental Phaseoleae EF024614	93	2			
	Potamogeton zosteriformis EF526358	86	Unc. Cercozoan EU567235	86				1
	Staurastrum pingue AJ428109	84	Unc. Eukaryote AJ829837	99			1	2

	Xanthidium armatum AJ428094	75	*Xanthidium armatum* AJ428094	75			2	
Alveolata								
Apicomplexa	*Colpodella edax* AY234843	89	Unc. Eukaryote AB252763	94			1	
	Cryptosporidium serpentis AF093501	87	Unc. Eukaryote AY331776	93				1
	Ophriocystis elektroscirrha AF129883	90	Unc. Eukaryote EF100310	91		3		
Ciliophora	*Parastrombidinopsis minima* DQ393786	90	*Parastrombidinopsis mínima* DQ393786	90			1	
	Parastrombidinopsis minima DQ393786	88-94	Unc. Eukaryote FJ765410 / AJ829840	94-97				3
	Tintinnopsis tubulosoides AF399111	99	Unc. Eukaryote FJ939124	99				1
	Varistrombidium sp. DQ811090	97	Unc. Eukaryote AY129053	97				1
Dinophyceae	*Amoebophrya* sp. AF472554	89	*Amoebophrya* sp. AF472554	89				2
	Amoebophrya sp.[*1]	85-94	Unc. Eukaryote[*2]	92-100	1	14	1	14
	Cachonina sp. AB183639 / AF033865	82-88	Unc. Alveolate DQ186536	84-100	1		1	
	Ceratium furca AJ276699	99	Unc. Eukaryote EF526910	99				1
	Duboscquella sp. AB295041	86-93	Unc. Eukaryote EF527151 / AY665057 / EU333031	91-99			5	8
	Gymnodinium aureolum DQ779991	99	*Gymnodinium aureolum* DQ779991	99			1	1
	Gymnodinium aureolum DQ779991	88	Unc. Eukaryote AY665030	98			10	10
	Gymnodinium catenatum DQ779990	93	Unc. Eukaryote FJ785963	96				2
	Gymnodinium sp. EF492493	78-99	*Gymnodinium* sp. EF492493	78-99			14	1
	Gymnodinium sp. AF274260	96	Unc. Eukaryote AY331745	99				1
	Gyrodinium helveticum AB120004	96	Unc. Eukaryote AY664972	98			1	
	Gyrodinium fusiforme AB120002	96-100	Unc. Eukaryote EF527094 / EF527101	96-99		1	7	
	Gyrodinium galatheanum AF274262	94	Unc. Eukaryote AY664962	98				1
	Heterocapsa niei EF492499	93	Unc. Eukaryote EF527111	95			1	
	Heterocapsa rotundata AF274267	98	*Heterocapsa rotundata* AF274267	98	2	1		
	Karlodinium micrum EF492490	84	*Karlodinium micrum* EF492490	84			4	
	Karlodinium micrum EF492506	89	Unc. Eukaryote AY789781	98			3	
	Lessardia elongata AF521100	86-88	Unc. Eukaryote[*2]	97-99		2		5
	Peridinium quinquecorne AB246746	96	Unc. Eukaryote FJ221800	96				1
	Polarella glacialis AY179607 / AF099183	81-97	Unc. Eukaryote DQ310254 / AJ402340	87-99		2	3	
	Prorocentrum mexicanum Y16232	99	*Prorocentrum mexicanum* Y16232	99		1		
	Prorocentrum triestinum EF492512	96	Unc. Eukaryote EF527089	99				1
	Pyrophacus steinii AY443024	80	*Pyrophacus steinii* AY443024	80	2			
	Roscoffia capitata AF521101	86-89	Unc. Eukaryote AY665030 / AB275022 / EU785270	86-99	9		4	8
	Warnowia sp. FJ947040	96	Unc. Eukaryote AY664877	98				1
Stramenopiles								
Bacillariophyta	*Attheya septentrionalis* AY485517	86-88	Unc. Eukaryote DQ344759	94-97				2
	Fragilariopsis cylindrus EF140624	99	*Fragilariopsis cylindrus* EF140624	99	1	2	2	
	Minutocellus sp. AY485520	97-99	Unc. marine Eukaryote FJ221912	97-99	11	3	35	2
	Pseudo-nitzschia pungens U18240	98	Unc. Eukaryote EU182819	98		2		
	Skeletonema japonicum DQ396518	98	*Skeletonema japonicum* DQ396518	98		1	2	

Chrysophyceae	*Paraphysomonas imperforata* EF432519	98	Unc. Eukaryote AY789782	98				1
Oomycetes	*Achlya bisexualis* M32705	85	*Achlya bisexualis* M32705	85		1		
	Haliphthoros sp. AB178865	93	*Haliphthoros* sp. AB178865	93			2	
	Lagenidium myophilum AB284577	93	*Lagenidium myophilum* AB284577	93			1	
Labyrinthulida	*Aplanochytrium kerguelense* AB022103	86-89	Unc. Eukaryote AY129068	93-96				1
	Aplanochytrium stocchinoi AJ519935	91-97	Unc. Labyrinthulid FJ800649 / FJ800598	91-97			7	6
	Labyrinthuloides minuta L27634	89	Unc. Eukaryote AB191425	97		1		
	Labyrinthuloides yorkensis AF265333	89-99	Unc. Eukaryote DQ103777 / AY129068	96-99	1	1	2	
	Oblongichytrium sp. AB290575	89	Unc. Eukaryote AY665006	90		1		
	Schizochytrium aggregatum AF265336	86	Unc. Eukaryote DQ344759	94				1
	Thraustochytriidae sp. DQ367047	88-90	Unc. Eukaryote EF526909 / 504337	92-95		1	2	
Hyphochytriomycetes	*Hyphochytrium catenoides* AF163294	89	Unc. Eukaryote	96				2
	Rhizidiomyces apophysatus AF163295	89-91	Unc. Eukaryote EF526909 / EU162648	91-96	1		6	
Pelagophyceae	*Coccoid pelagophyte* U40927	92	*Coccoid pelagophyte* U40927	92				1
	Pelagomonas calceolata EF455763	100	*Pelagomonas calceolata* EF455763	100				4
Unclassified	*Pirsonia diadema* AJ561114	93	Unc. Eukaryote AY331765	93				1
Rhizaria								
Acantharea	*Chaunacanthid* sp. AF018158	95	Symphyacanthid AF063242.1	95			1	
Cercozoa	*Allas* sp. AY268040	90	Unc. Eukaryote FJ222163	89			1	
	Phagomyxa bellerocheae AF310903	92	*Phagomyxa bellerocheae* AF310903	92		2		
	Cercomonas sp. AF411266	88	Unc. Cercozoan EU785280	96	5	3		
	Cercozoa sp. EU709238	90	Unc. Cercozoan AY620336	92		1		
	Protaspis obliqua FJ824122	90-96	Unc. Eukaryote FJ985885 / AY620309	93-99	4		1	
	Protaspis sp. FJ824125	92-97	Unc. Eukaryote AY885064 / DQ234284	92-98		3	8	2
Gromiidae	*Gromia oviformis* AJ457811 / AJ457812	92-97	*Gromia oviformis* AJ457811 / AJ457812	92-97		2	1	
Foraminifera	*Ammonia beccarii* U07937	81-96	Unc. Eukaryote AY179972 / EF100299	82-100		1	1	
Polycystinea	*Dictyocoryne profunda* AB101540	93	Um. Eukaryote EU333071	99				4
Unclass. Rhizaria	*Paradinium poucheti* EU189031	84	Unc. Eukaryote EU798716	83			1	1
Cryptophyta	*Teleaulax amphioxeia* AJ421146	99	Unc. eukaryote DQ310323	98		1		
Unclass. Eukaryotes								
Cryomonadida	*Cryothecomonas aestivalis* AF290539	98	Unc. Eukaryote AY885052	99	1		1	1

*1 - AY208893, AY775285, AF0695161, AF472554;

*2 - FJ785975, AY129041, EF172843, EF172848, EU793779, AY665001, EU793261, EU785256, EU793852, EU333103, EF172945, AY129038, AB252763, DQ647524.
